# Metabolic Reprogramming of the Host Cell by Human Adenovirus Infection

**DOI:** 10.3390/v11020141

**Published:** 2019-02-08

**Authors:** Martin A. Prusinkiewicz, Joe S. Mymryk

**Affiliations:** 1 Department of Microbiology and Immunology, Western University, London, ON N6A 3K7, Canada; mprusink@uwo.ca; 2 Department of Otolaryngology, Head & Neck Surgery, Western University, London, ON N6A 3K7, Canada; 3 Department of Oncology, Western University, London, ON N6A 3K7, Canada; 4 London Regional Cancer Program, Lawson Health Research Institute, London, ON N6C 2R5, Canada

**Keywords:** human adenovirus, E1A, E4ORF1, metabolism, glycolysis, glutaminolysis, Warburg effect, MYC, HAdV5, HAdV36

## Abstract

Viruses are obligate intracellular parasites that alter many cellular processes to create an environment optimal for viral replication. Reprogramming of cellular metabolism is an important, yet underappreciated feature of many viral infections, as this ensures that the energy and substrates required for viral replication are available in abundance. Human adenovirus (HAdV), which is the focus of this review, is a small DNA tumor virus that reprograms cellular metabolism in a variety of ways. It is well known that HAdV infection increases glucose uptake and fermentation to lactate in a manner resembling the Warburg effect observed in many cancer cells. However, HAdV infection induces many other metabolic changes. In this review, we integrate the findings from a variety of proteomic and transcriptomic studies to understand the subtleties of metabolite and metabolic pathway control during HAdV infection. We review how the E4ORF1 protein of HAdV enacts some of these changes and summarize evidence for reprogramming of cellular metabolism by the viral E1A protein. Therapies targeting altered metabolism are emerging as cancer treatments, and similar targeting of aberrant components of virally reprogrammed metabolism could have clinical antiviral applications.

## 1. Introduction

Viruses are obligate intracellular parasites. As such, they are critically dependent upon energy and substrates obtained from the infected host cell. Human adenoviruses (HAdVs) are double-stranded DNA tumour viruses with a genome of approximately 36 kilobase pairs. There are approximately 90 specific types distributed across 7 species, termed A through G, based on genetic and biological characteristics (Table 1). HAdVs exhibit a variety of tissue tropisms, often dependent on HAdV type, including preference for respiratory, gastrointestinal, ocular, or renal tissues [1,2]. HAdVs generally cause acute, lytic infections with a replicative cycle of typically several days between exposure and production of new viruses in quiescent epithelial cells. In one round of infection, a single infectious virion leads to the production of thousands of infectious progeny. Viral replication requires the substrates and energy provided by the host cell, and an optimized environment within the virus infected cell ensures maximal HAdV progeny production. HAdV proteins interact with host-cell proteins to modify cellular functions, creating amenable conditions for virus replication and virion production regardless of any pre-existing cell state.

The adenovirus genome is organized into early and late regions, corresponding to the temporal kinetics of transcription of these regions [3]. The early region consists of multiple transcription units, termed E1A, E1B, E2A, E2B, E3 and E4 [1]. The products of the E1A transcription unit function to control transcription of viral genes, as well as modify host-cell gene expression to benefit viral reproduction [4]. The E1B products modulate host-cell proliferation, apoptosis and assist with viral replication [3]. The products from the E2 transcription units are primarily involved in viral DNA replication [3]. The E3 transcription unit encodes viral proteins that subvert host immune responses [3]. The E4 transcription unit is comprised of 7 open reading frames (ORFs), the products of which act to modulate cellular function and assist with viral DNA replication and RNA processing [5]. There is a single late transcription unit that is alternatively spliced to yield five groups of mRNAs termed L1 through L5. Late mRNAs encode products that are viral structural proteins or contribute to virion production [3]. Other transcription units expressed during intermediate timepoints of infection, such as pIX and IVa2, perform structural functions or play a role in viral packaging [3]. In addition, some of the viral proteins are oncoproteins capable of inducing cancer-like phenotypes. For example, the HAdV E1A oncoprotein is capable of transforming many cell types [6] in conjunction with a second oncoprotein, such as RAS, or the HAdV E1B oncoproteins. E4ORF1, another HAdV oncoprotein [7], can influence host-cell metabolism, which will be discussed extensively in this review. Many HAdV types can oncogenically transform rodent cells [6,7], but HAdV is not currently associated with any human cancer, possibly due to the lytic nature of HAdV infection. However, some viruses with a lytic cycle, such as the γ-herpesviruses Epstein-Barr virus (EBV) and Kaposi’s sarcoma-associated herpes virus (KSHV), are oncogenic and expression of their lytic genes can contribute to oncogenesis [8,9,10,11]. A recent whole genome analysis of multiple tumour types for viral signatures indicated that HAdV DNA may be especially prevalent in kidney, breast, prostate, and head and neck tumours, suggesting a previously unsuspected causal relationship [12]. Understanding how HAdV reprograms cellular metabolism is important, as it yields insight into the functions of various viral oncoproteins and reveals parallels between virally induced metabolic changes and cancer metabolism. This review focuses on the alterations made by HAdV to host cell metabolism. Recent metabolomic and proteomic studies will be reviewed and mechanisms by which the viral oncoproteins E4ORF1 and E1A alter metabolism will be discussed.

## 2. Glycolysis and the Warburg Effect

**Figure 1:**
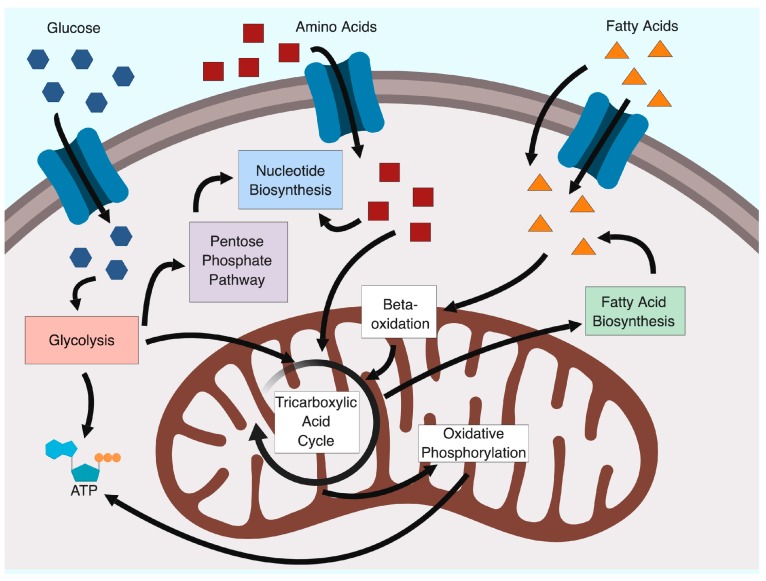
Viruses co-opt many cellular metabolic pathways to satisfy their metabolic requirements. These pathways include those used for energy production, primarily glycolysis and oxidative phosphorylation, and macromolecule production, such as for the synthesis of nucleotides or fatty acids. Created with BioRender.

Cellular energy production typically begins with the conversion of glucose to pyruvate through glycolysis. Pyruvate is funnelled to the tricarboxylic acid (TCA) cycle to load electrons onto various coenzymes that can be utilized in the electron transport chain to convert ADP to ATP (Figure 1). However, many metabolites within glycolysis and the TCA cycle can be utilized in other pathways to generate precursors for macromolecules required for viral replication. For example, intermediates of glycolysis can be funnelled into the pentose phosphate pathway (PPP) to generate ribose, the sugar backbone of nucleotides (Figure 1).

**Figure 2:**
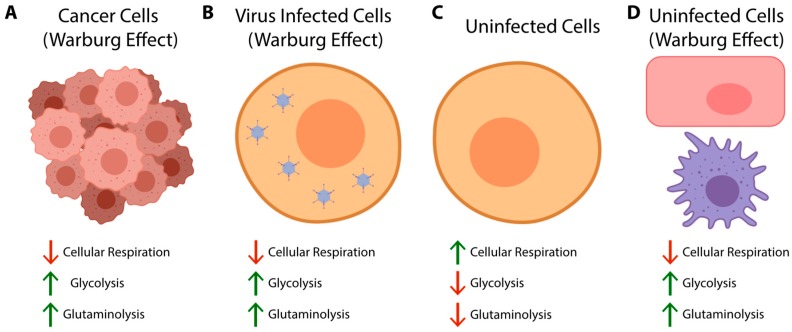
Both cancer cells (**A**) and virus infected cells (**B**) often exhibit a characteristic metabolic phenotype known as the Warburg effect. This phenotype is associated with an increase in cellular glycolysis and a concurrent decrease, albeit not a complete reduction, of cellular respiration despite the availability of ample oxygen. In contrast, healthy, uninfected cells (**C**) preferentially utilize cellular respiration over glycolysis as the main ATP generating pathway. Glutaminolysis is also less active in uninfected non-transformed cells. However, there are uninfected cells (**D**) that preferentially utilize the Warburg effect. For example, endothelial cells consistently have a Warburg effect-like metabolic phenotype [19]. Activated immune cells, such as effector T cells, activated macrophages, and activated dendritic cells, also shift to a Warburg effect-like metabolic phenotype [20]. Created with BioRender.

Typically, cells prefer the slower, but more energetically productive electron transport chain as the main source of cellular energy over glycolysis. Glycolysis proceeds rapidly, but produces much less energy. However, under certain conditions, cells appear to utilize glycolysis over cellular respiration, despite the presence of ample oxygen. This is known as the Warburg effect (Figure 2), and was first observed in cancer cells [13,14,15]. It is becoming increasingly appreciated that many viruses reprogram cellular metabolism in a similar manner (Figure 2). For example, DNA tumour and tumour-associated viruses, such as human papillomavirus (HPV), KSHV, EBV, human cytomegalovirus (HCMV) and HAdV, are all noted to increase host cell glycolytic activity (reviewed in [16,17]). Some single-stranded RNA viruses, such as poliovirus, dengue virus, hepatitis C virus (HCV) and influenza A virus have also been noted to increase glycolysis [16,17]. In addition, the Warburg effect is more complex than initially appreciated, as it is commonly accompanied by glutaminolysis [18] (Figure 2), which includes the utilization of glutamine as a substrate in the TCA cycle. This means that cells exhibiting the Warburg effect still utilize cellular respiration, albeit to a lesser extent than cells with a normal metabolic phenotype.

## 3. The Earliest Observations of Metabolic Changes due to HAdV Infection

**Figure 3:**
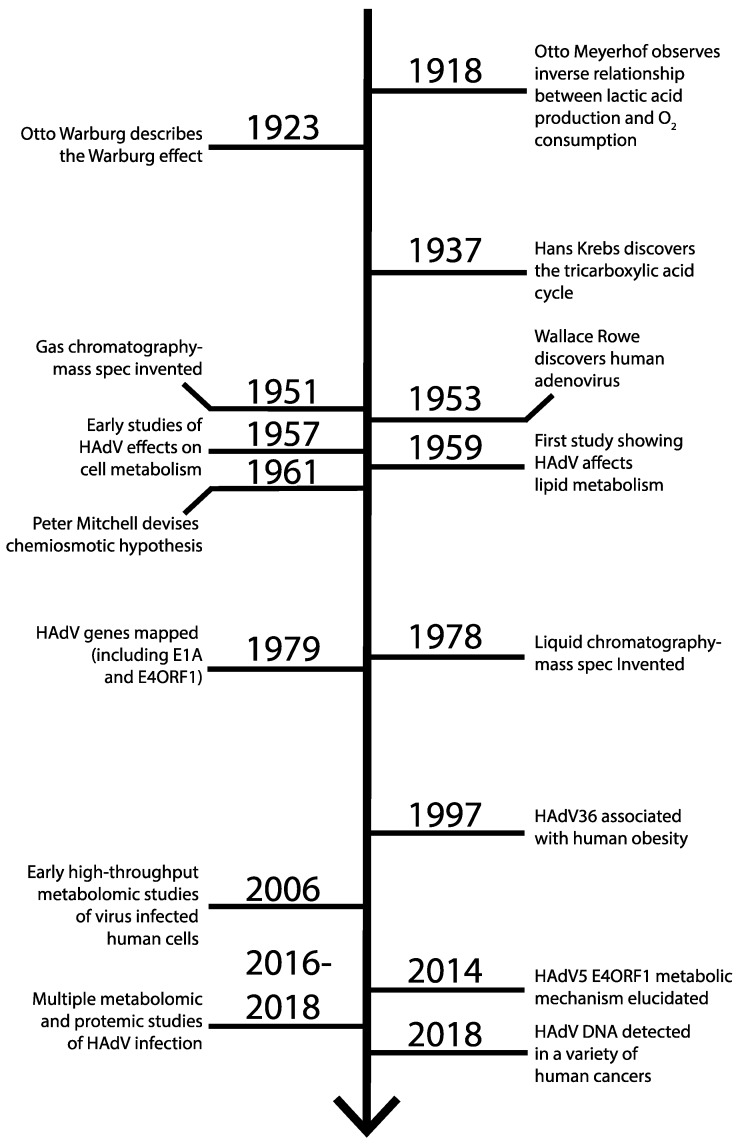
Key relevant advances in metabolism research, adenovirus research and technology that allowed for contemporary high-throughput studies on the effect of HAdV infection on cellular metabolism. The early 20th century featured many insights into the basics of cellular metabolism. The discovery of HAdV and early studies on the effect of HAdV on host-cell metabolism were performed in the 1950s. Little further research on the influence of HAdV on cellular metabolism was performed until the 21st century, when advances in metabolomic, proteomic and genomic technology allowed for thorough study of host-cell metabolic changes.

Shortly after adenoviruses were discovered in 1953 by Wallace Rowe and colleagues [21], the effects of HAdV infection on metabolism were explored in cell culture (Figure 3). During these early investigations, similarities in metabolic reprogramming between HAdV types were recognized [22,23,24]. For example, HAdV species B type 7 (HAdVB-7) (Table 1) infection of HeLa cells (Table 2) was noted to exhibit increased lactic acid production, likely due to an increase in glucose utilization, when compared to uninfected HeLa cells [24]. This increased lactic acid production corresponded to a 2-fold increase in lactate dehydrogenase activity in infected cells [24]. In addition, the TCA cycle was necessary for HAdVB-7 replication, as inhibition of this pathway with sodium fluoroacetate decreased viral titre by 300× [24], serving as a precursor to the subsequent recognition of the importance of glutamine and glutaminolysis for viral replication [25,26].

The upregulation of nucleotide biosynthesis by HAdV infection was also discovered in the early years of HAdV research. In 1964, HAdV species C type 5 (HAdVC-5) (Table 1) was found to cause a 2- to 3-fold increase in aspartate transcarbamylase activity at 18 h post infection (hpi) in HeLa cells [27]. Aspartate transcarbamylase activity is a function of the first enzyme in the pyrimidine biosynthesis pathway, carbamoyl phosphate synthetase-aspartate transcarbamylase-dihydroorotase (CAD) [28]. In another paper from 1971, increased cellular lipid metabolism, primarily triglyceride production, was associated with HAdVC-5 infection of human embryonic kidney (HEK) cells (Table 2) [29]. As expected, these lipids were not incorporated into the HAdVC-5 structure, since HAdV is a non-enveloped virus [29]. As this increase in lipid metabolism could similarly be induced by a UV-inactivated virus, a structural feature of the virus was possibly responsible for the upregulation [29]. Indeed, exposure of the cell to purified HAdV structural proteins indicated that the penton and penton-base proteins, but not the fiber or hexon proteins, were at least partially responsible for this increase in lipid metabolism [29].

Many years passed between these initial observations and advances in high-throughput metabolomics technology that allowed for the first metabolomics study of virus-infected human cells in 2006 [30]. Indeed, thorough metabolomic studies of HAdV infected cells began in 2016 [31]. These metabolomic studies of HAdV infected cells will be discussed in the next section. Important relevant discoveries in metabolism, HAdV virology and high-throughput metabolomics technologies are summarized in the timeline depicted in Figure 3.

## 4. Metabolomic and Proteomic Analyses of Adenovirus Infection

Since 2016, many high-throughput metabolic studies on HAdV infected cells have been performed. Key studies will be summarized in this section. Recent genomic and proteomic studies of HAdV infected cells in the context of host-cell metabolic changes will also be summarized.

An investigation using ^1^H-NMR spectroscopy looked for changes in 35 metabolite concentrations in HEK293 (Table 2) and human amniocyte derived 1G3 cells (Table 2) during infection with E1-region deleted HAdVC-5 [31]. Although cells were infected with an E1-region deleted HAdVC-5, this study essentially measured the effects of wild type HAdVC-5 infection as both HEK293 and 1G3 cells effectively complement the viral defect by expressing the E1A and E1B regions of HAdVC-5. The main finding of this study was that glucose consumption doubles and lactate secretion increases 4-fold compared to respective uninfected cells [31].

**Figure 4:**
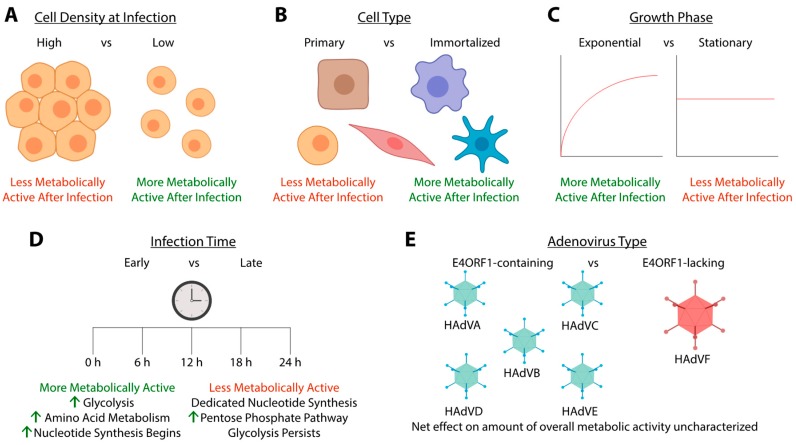
Factors influencing host-cell metabolism with HAdV infection. (**A**) HAdV induced changes in cellular metabolism are less drastic in cells infected at a high cellular density in comparison to cells infected at a low cellular density [31]. (**B**) Cell type can influence the metabolic reprogramming enacted by HAdV. Primary cells are usually slower growing than immortalized cells, which is reflected in a lower metabolic rate. Although metabolism is changed across various cell types upon HAdV infection [31], the rate of that change is likely faster in immortalized cells and contributes to rapid viral replication in immortalized cells [42,43,44]. However, even among immortalized cells, those with a phenotype more closely resembling the Warburg effect appear primed for HAdV replication and experience more drastic metabolic changes than immortalized cells with a metabolic phenotype reliant on oxidative phosphorylation [45]. (**C**) Growing and dividing cells infected with HAdV show more drastic metabolic changes than infected quiescent cells [46]. (**D**) The metabolic profile of HAdV infected cells changes throughout the course of infection [47]. Initially, HAdV infected cells typically exhibit upregulated glycolysis, amino acid metabolism and nucleotide biosynthesis pathways [47]. Towards the later stages of infection, HAdV infected cells still perform glycolysis, but the majority of metabolic activity is directed towards nucleotide biosynthesis and an upregulation of the pentose phosphate pathway (PPP) occurs [47]. (**E**) Different HAdV types regulate metabolism through mechanisms related to the functions of HAdV E4ORF1 proteins. Some HAdV types (e.g., HAdVF-40) do not have E4ORF1 and clearly rely on other HAdV proteins to regulate metabolism [48]. E1A, which also varies among HAdV types, is another potential regulator of cell metabolism during infection [49,50,51]. Created with BioRender.

This study also examined the effects of cell density on metabolic changes induced by HAdVC-5 infections. Lower cell density at infection was associated with better HAdVC-5 production and more extreme metabolic responses [31]. Interestingly, glutamine exhaustion was limiting for HAdV replication, especially at higher cell densities [31]. In addition, this study explored whether glutamine replenishment and pH control with cells grown in a bioreactor yielded a similar metabolic phenotype upon HAdVC-5 infection. The results of these experiments suggest that 1G3 cells are less reliant on glutamine during infection [31]. In short, cellular density at infection had significant effects on metabolism [31] (Figure 4A). While glucose consumption trends were similar in both 1G3 and HEK293 cells, consumption and production of other metabolites (Supplementary Table S1) could vary with cell type (Figure 4B) and growth phase [31] (Figure 4C), especially when the slower replication rate of HAdV in primary cells is considered [42,43,44].

Another study measured the metabolic flux of [1,2-^13^C] glucose and [U-^13^C] glutamine in 1G3 cells infected with E1-deleted HAdVC-5, conditions which again essentially recapitulated a wild type HAdVC-5 infection [46] (Supplementary Table S1). In 1G3 cells infected with HAdVC-5 during exponential growth, glycolysis was upregulated by 17%, as evidenced by higher ^13^C incorporation in glycolytic intermediates than TCA cycle intermediates, with a corresponding 4-fold increase in PPP [46]. Lactate production also increased with glucose production, as observed in other studies [26,31]. Increases in other metabolites, such as amino acids, under these conditions are shown in Supplementary Table S1.

That study also reported an interesting 2-fold increase in acetyl-CoA production from citrate [46], a process associated with fatty acid biosynthesis. Increased lipid biosynthesis is a logical requirement for enveloped viruses, and both enveloped and non-enveloped viruses can increase lipid biosynthesis for the expansion of membrane bound viral replication compartments (reviewed in [52,53]). However, HAdV replication compartments are located in the nucleus and are not surrounded by a membrane [54]. This leaves the reasons for the potential increase of lipid biosynthesis during HAdV infection unclear.

While overall metabolic activity was increased in 1G3 cells infected with HAdVC-5 during stationary phase, which was induced by a combination of cell confluency and serum deprivation for 36 h, these metabolic changes were different compared to 1G3 cells infected during exponential growth [46]. Metabolic changes that occurred in infected stationary 1G3 cells included a 1.5-fold increase in glutamine catabolism [46], which serves to replenish TCA cycle intermediates when they might be limited due to the conversion of pyruvate to lactate by the Warburg effect [55]. A corresponding 1.5-fold increase in the TCA cycle itself also occurred in infected stationary 1G3 cells [46]. Glucose consumption in HAdVC-5-infected stationary 1G3 cells increased, with a corresponding increase in lactate production [46]. Production of specific amino acids also increased (Supplementary Table S1) [46]. An increase in acetyl-CoA production from citrate was observed with HAdVC-5 infection of growth arrested 1G3 cells [46]. However, the PPP was not stimulated and overall HAdVC-5 production decreased 4-fold when compared to exponentially growing HAdVC-5-infected 1G3 cells [46].

The metabolic state of HAdV infected cells also changes longitudinally (Figure 4D). A study analyzing changes in cellular protein expression of HAdV species C type 2 (HAdVC-2) (Table 1) infected growth-arrested IMR-90 cells (Table 2) at 6, 12, 24 and 36 hpi identified a variety of metabolism related proteins with differential expression throughout infection. Early during infection, starting at 6 h and persisting through to 12 hpi, proteins encoding enzymes involved in glycolysis and *de novo* purine and pyrimidine synthesis were upregulated [47]. The upregulation of glycolytic and nucleotide biosynthesis proteins persisted through to the later 24 and 36 hpi time points [47]. Unique to the 6 and 12 h time points was an upregulation of proteins involved in glutathione metabolism [47] (Supplementary Table S2), which is responsible for detoxifying reactive oxidative species, perhaps generated as a result of virus infection [56]. An analysis of upregulated pathways indicated that at the earliest time point (6 hpi) serine glycine biosynthesis (Supplementary Table S2), and mannose metabolism (Supplementary Table S2) were upregulated [47]. The serine glycine biosynthesis pathway converts 3-phosphoglycerate into serine, and eventually glycine [57], which could account for some of the increased intracellular amino acid concentrations noted in the two studies mentioned above [25,46]. Mannose metabolism is responsible for contributing to protein glycosylation [58,59]. Later, at 12 hpi, proteins involved in fructose galactose metabolism (Supplementary Table S2) were upregulated and likely contribute to the upregulated glycolysis occurring at all time points [47]. There were also two enzymes from the PPP that were upregulated at 12 hpi (Supplementary Table S2). At 24 hpi, most proteins involved in the PPP were upregulated, although the authors did not find any changes in mRNA expression for PPP genes [47]. This may be due to changes in expression based on cell type and/or differences in infection timing between these two studies. At 24 hpi, a few proteins involved in serine glycine biosynthesis continued to be upregulated (Supplementary Table S2), which could contribute to the production of glycine used for purine biosynthesis [57].

In the same study, an analysis of putative transcription factors regulating the expression of metabolic genes during HAdV infection indicated that MYC was significantly upregulated at all time points [47]. Another transcription factor potentially responsible for the upregulation of metabolic genes in HAdV infection was E2F1 [47]. The ATF/CREB family of transcription factors were also upregulated [47]. ATF/CREB transcription factors are responsible for upregulating metabolism [60] and are also known targets of E1A [61,62,63]. Finally, the transcription factor NRF2, which has metabolism associated regulatory functions [64], was potentially responsible for the expression of a wide variety of metabolic genes at all time points during HAdV infection [47]. The metabolic functions of NRF2 include inhibiting lipogenesis, activating fatty acid oxidation, influencing the PPP, as well as enhancing purine biosynthesis and NADPH production [64].

Another study compared the effects of infection with HAdVC-5, wild-type HAdV species B type 11p (HAdVB-11p) (Table 1), and an oncolytic HAdV, enadenotucirev (EnAd, formerly ColoAd1), on metabolism of A549 cells (Table 2) and SKOV3 ovarian carcinoma cells (Table 2) [45]. HAdV infection increased glycolysis and glutaminolysis [45], as expected [25,26,46,47]. However, counterintuitively, the authors found that inhibiting glycolysis with 2-deoxyglucose (2DG) or limiting glucose availability increased viral genome replication and packaging efficiency in both A549 cells and SKOV3 cells [45]. Inhibition of glycolysis in SKOV3 cells, which, unlike A549 cells, exhibit a metabolic phenotype that does not resemble the Warburg effect [65], also increased the speed of EnAd and HAdVB-11p viral replication and progeny production [45]. Glucose limitation is hypothesized to be beneficial to the expression of late proteins during HAdV infection, which could explain why HAdV progeny production was increased with 2DG [45]. These results were maintained when viral replication was measured in SKOV3 cells lacking functional endogenous glycolysis, in primary human ascites cells and an in vivo xenograft mouse model treated with 2DG [45]. Furthermore, A549 cells grown in glutamine limiting conditions had a 1 × 10^5^-fold reduction in the production of infectious EnAd or HAdVB-11p virions [45]. These results indicate that glycolysis is expendable, and perhaps even detrimental to viral replication at higher levels. However, HAdV infected cells generally require glutamine, but the extent to which glutamine is required may vary with HAdV type, as HAdVC-5 did not appear to have a similar dependence [45].

When a variety of other TCA cycle intermediates were supplemented to glutamine limited A549 or SKOV3 cells infected with EnAd, only α-ketoglutarate, not oxaloacetate or pyruvate, was able to completely rescue HAdV virion production [45]. This suggests that rather than wholly being used to fuel the TCA cycle, glutamine may also be broken down to α-ketoglutarate, which is used for production of other macromolecules required for viral replication, including amino acids and/or lipids.

An LC-MS proteomic study of A549 cells infected at confluency with HAdV species F type 40 (HAdVF-40) (Table 1) and examined at 30 hpi indicated that 206 host-cell proteins were upregulated and 130 host-cell proteins were downregulated by infection [48]. Many of these were involved in metabolism and energy production pathways. Specifically, these included glycolysis, the TCA cycle, cellular respiration, beta-oxidation, the PPP, and amino acid metabolism [48]. Interestingly, the authors observed higher mitochondrial activity in HAdVF-40 infected cells [48]. In addition, two glycolytic proteins upregulated by HAdVC-5 infection, HK2 and PFKM, were not induced in HAdVF-40 infected cells [48]. HAdVF-40 does not encode an E4ORF1 equivalent, which may explain why these two specific glycolytic enzymes are not upregulated by HAdVF-40 infection [48]. This also suggests that, despite E4ORF1 being the only HAdV protein currently implicated in transcriptionally regulating metabolism upon infection, HAdV proteins other than E4ORF1 contribute to transcriptional regulation of host-cell metabolism gene expression (Figure 4E).

## 5. E4ORF1 Positively Regulates Glycolysis and Glutamine Catabolism

The only concrete mechanism by which adenovirus is currently known to regulate host-cell metabolism is through its E4ORF1 protein [25,26]. E4ORF1 is a viral oncoprotein that can transform rat embryonic fibroblasts through its C-terminal PDZ-binding domain [7,66]. This PDZ-binding domain binds host-cell PDZ domain proteins and mediates the activation of PI3K and AKT, leading to oncogenic transformation [67]. However, the ability of E4ORF1 to regulate glycolysis is independent of this C-terminal domain [26].

**Figure 5:**
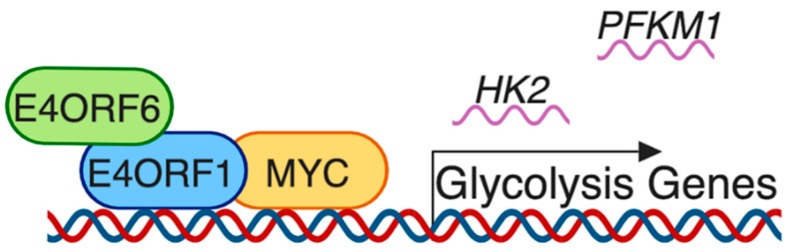
Schematic of how E4ORF1 contributes to MYC-regulated transcription of genes involved in glycolysis according to Thai et al. [26]. E4ORF1 binds to MYC, enhancing the transcriptional activity of MYC, leading to increased transcription of metabolic genes such as *HK2* and *PFKM1*. E4ORF1 can also bind glycolytic genes, which may be how E4ORF1 brings MYC into proximity of these target genes. E4ORF6 appears to play a scaffolding role and enhances E4ORF1 binding to MYC, although E4ORF6 does not appear to bind MYC or glycolytic genes itself [26]. Created with BioRender.

Thai et al. observed that MCF10A breast epithelial cells (Table 2) infected with wild type HAdVC-5 had increased glucose consumption and increased lactate production compared to uninfected cells [26] (Supplementary Table S1). These metabolic changes were accompanied with decreased oxygen consumption and presumably less oxidative phosphorylation compared to uninfected cells [26]. Thai et al. found that cells infected with a non-replicating Δ*E4* HAdVC-5 mutant did not have increased glycolysis or decreased oxidative phosphorylation [26]. When MCF10A cells were engineered to express the adenovirus E4 region alone, glycolysis was increased, as indicated by increased glucose consumption and lactate production [26]. However, oxidative phosphorylation was not affected, as there was no change in oxygen consumption in these cells [26]. E4ORF1 was identified to be the viral protein responsible for these metabolic changes, but these changes were enhanced in the presence of E4ORF6, which is known to have a stabilizing effect on E4ORF1 [26]. Microarray with gene set enrichment analysis (GSEA) in MCF10A cells constitutively expressing E4ORF6 and transfected with either E4ORF1 or an empty vector identified genes regulated by MYC as being particularly upregulated by E4ORF1 [26]. This MYC upregulation agrees with another high throughput study looking at transcription factors regulated by HAdV infection [47]. Chromatin immunoprecipitation quantitative polymerase chain reaction (ChIP-qPCR) analysis indicated that MYC binding to glycolytic genes was increased in E4ORF1 transfected cells and E4ORF1 was also found bound to some glycolytic genes [26] (Figure 5). E4ORF1 formed a physical interaction with MYC, supported by E4ORF6, and this increased MYC localization to the nucleus [26]. These changes corresponded to increased *HK2* and *PFKM1* mRNA levels in E4ORF1-expressing cells [26]. In agreement with this, A549 cells infected with HAdVF-40, which does not contain E4ORF1, do not exhibit elevated levels of HK2 or PFKM protein [48].

Thai et al. identified that E4ORF1 was responsible for regulating metabolic changes, as a point mutation in this viral protein, D68A, abrogated all of the metabolic changes associated with E4ORF1 in both vector transfection and mutant virus infection [26]. shRNA knockdown of MYC also abrogated the glycolytic metabolic changes associated with E4ORF1 during HAdV infection [26]. In addition, MYC knockdown decreased viral titre, providing evidence that metabolic changes do indeed enhance virus yield during infection [26]. Interestingly, viral titre from cells infected with E4ORF1-D68A mutant HAdVC-5 was only lower in infected HeLa cells, but not in infected MCF10A cells. Thai et al. attribute this to the higher glycolytic activity of MCF10A cells [26].

Finally, increased nucleotide metabolism is one of the consequences of upregulated glycolysis. Thai et al. traced carbon from ^13^C-labelled glucose to nucleotides during wild type HAdVC-5 infection in normal human bronchial epithelial cells (NHBE) (Table 2). Increased ^13^C incorporation into nucleotides did not occur during infection with HAdVC-5 E4ORF1-D68A [26]. Correspondingly, transcripts of *RPIA* and *RPE*, two genes involved in the non-oxidative branch of the PPP, were only upregulated in cells infected with wild type HAdVC-5, but not HAdVC-5 E4ORF1-D68A [26]. This upregulation of the PPP with an increase in glycolysis matches the observations in another high throughput metabolomics study [46]. However, a second high throughput metabolomics study found no changes in mRNA levels for any PPP genes [47].

In a follow-up study, HAdVC-5 infection of NHBE was associated with increased glutamine consumption during early infection (Supplementary Table S1), which occurred at approximately 8 to 12 hpi [25]. This increased consumption was abrogated by shRNA knockdown of MYC, or infection with the non-MYC binding E4ORF1-D68A mutant adenovirus [25]. miRNAs miR-23a and miR-23b, which are associated with decreased glutaminase expression, were also downregulated starting at 90 min post wild type HAdVC-5 infection [25]. LC-MS/MS U-^13^C_5_-glutamine labelling indicated that HAdVC-5 infected cells had a pattern of carbon labelling that corresponded to reductive carboxylation [25]. Reductive carboxylation is the carboxylation of α-ketoglutarate, produced from glutamine, to citrate. This citrate can be used to produce lipids from interconversion to acetyl-CoA or fuel the TCA cycle [68]. mRNA transcripts associated with reductive carboxylation were also upregulated with HAdVC-5 infection [25], but not in HAdVC-5 E4ORF1 D68A mutant infections [25].

Further emphasizing the importance of glutamine during HAdVC-5 infection, transcripts for glutamine transporter genes *ASCT2* and *LAT1* were higher in HAdVC-5 infected cells [25]. These transporters exchange glutamine for other amino acids. There were higher intracellular concentrations of both essential and non-essential amino acids in HAdVC-5 infected NHBE cells as compared to uninfected or HAdVC-5 E4ORF1-D68A mutant infected cells [25]. Increases in intracellular amino acid concentrations (Supplementary Table S1) matched what was observed in another high throughput metabolomics study [46]. Concentrations of amino acids likely increase to provide substrates required for virus replication. Another pathway associated with HAdVC-5 infection-induced glutamine metabolism is hexosamine biosynthesis [25], which produces UDP-GlcNAc. UDP-GlcNAc can be used for O-GlcNAc protein modification to alter the activity of metabolic enzymes, such as those involved in glycolysis [69]. The importance of glutamine for adenovirus replication is also emphasized by the ability of CD-839, an inhibitor of glutaminase, to reduce HAdVC-5 replication at least 80-fold [25]. However, whether any of these changes in glutamine metabolism are linked to HAdVC-5-infection induced decreases in oxidative phosphorylation remains to be explored.

## 6. Human Adenovirus 36 Influences Metabolism through E4ORF1

Despite the cellular metabolic changes enacted by HAdVC-5 E4ORF1 discussed above, no HAdV types are conclusively linked to any human metabolic disorders. Although some adenovirus types, such as HAdV species D type 36 (HAdVD-36) (Table 1) [70] and HAdV species A type 31 (HAdVA-31) (Table 1) [71,72], are prevalent in obese individuals, this may simply be due to the higher susceptibility of obese individuals to viral infections [73]. However, HAdVD-36 has been associated with metabolic changes in animal models, including mice, chickens and non-human primates. [74,75]. Interestingly, these metabolic effects are linked to the HAdVD-36 E4ORF1 protein. The downstream pathways affected by HAdVD-36 E4ORF1 have been studied in detail and are somewhat different from those affected by HAdVC-5 E4ORF1. However, commonalities still exist, which gives further insight into general HAdV E4ORF1 function. Considering that obesity is a risk factor for certain types of cancer [76], understanding how HAdVD-36 reprograms metabolism could yield insight into metabolic pathways that may also prime the cell for a cancer-like phenotype. In one example study, both HAdVD-36 infection or expression of its E4ORF1 protein alone increased glucose consumption (Supplementary Table S1) in 3T3-L1 adipocytes (Table 2) due to an increase in overall GLUT4 protein and phospho-AKT mediated translocation of GLUT4 to the plasma membrane [77].

Another study compared the effects of HAdVD-36 E4ORF1 transduction to the effects of HAdVC-5 E4ORF1 transduction a diabetes mouse model (*db/db*) and a diet-induced obesity mouse model [78]. HAdVD-36 E4ORF1 was able to improve glycemic control and enhance glucose disposal independently of insulin [78]. In addition, high doses of HAdVD-36 E4ORF1 lowered non-fasting blood glucose in wild type mice [78]. Similar effects on blood glucose levels were not seen with HAdVC-5 E4ORF1. Effects that were specific for *db/db* mice transduced with either HAdVC-5 E4ORF1 or HAdVD-36 E4ORF1 included decreased body weight in both transduced mice groups, combined with decreased food intake [78]. However, the HAdVC-5 E4ORF1 transduced mice did not have reduced blood glucose or glycemic control [78]. Also, the HAdVD-36 E4ORF1 transduced *db/db* mice had reduced insulin and no changes in liver or fat mass [78]. Serum levels of adiponectin, which regulates fatty acid beta-oxidation and glucose metabolism, were also lower in HAdVD-36 E4ORF1 transduced mice [78]. In diet-induced obese mice transduced with HAdVD-36 E4ORF1, a decrease in body weight, despite no change in food intake, occurred [78]. Also, in these mice, blood glucose decreased, glycemic control increased and liver weight increased [78]. In wild type mice transduced with a high dose of HAdVD-36 E4ORF1, lowered blood glucose, increased serum fatty acids and increased liver mass were observed [78]. These changes did not occur in wild type mice transduced with HAdVC-5 E4ORF1 [78]. In the liver of mice transduced with HAdVD-36 E4ORF1, fatty acid concentrations were increased, while glycogen concentrations were decreased [78]. Liver fatty acid concentrations also increased in HAdVC-5 E4ORF1 transduced mice [78]. To summarize, in *db/db* diabetic mice and diet-induced obese mice, the HAdVD-36 E4ORF1 protein alone improved glycemic control and lowered non-fasting blood glucose, while the HAdVC-5 E4ORF1 protein did not (Table 3). This suggests that HAdVD-36 E4ORF1 may actually counteract the obesogenic effects otherwise observed during HAdVD-36 infection of mice.

While the above changes occurred systemically in *db/db* mice and obese mice, changes in liver gene expression were also examined in the study described above using RT-qPCR [78]. Gene expression changes were examined in both *db/db* mice and diet-induced obese mice. These changes revealed more about the possible mechanism by which E4ORF1 regulates metabolism of host-cells. *db/db* mice transduced with HAdVD-36 E4ORF1 had upregulated glycolysis-related transcripts (Supplementary Table S2) [78]. *PDK4* transcript, which encodes a kinase responsible for inhibiting pyruvate dehydrogenase and therefore inhibiting the TCA cycle following glycolysis, was higher in *db/db* mice transduced with HAdVC-5 E4ORF1 [78]. Transcripts related to fatty acid synthesis were downregulated in both HAdVD-36 E4ORF1 or HAdVC-5 E4ORF1 transduced *db/db* mice (Supplementary Table S2) [78].

In the diet-induced model of obesity, mice transduced with HAdVD-36 E4ORF1 had an upregulation of the glycolytic genes in the liver (Supplementary Table S2) [78]. Upregulation of other metabolic mRNAs was observed in the livers of HAdVD-36 E4ORF1 transduced diet-induced obese mice (Supplementary Table S2) [78]. In diet-induced obese mice transduced with HAdVC-5 E4ORF1, only the fatty acid metabolism related gene *SCD1* was uniquely upregulated [78]. *G6PD*, involved in the PPP, was downregulated in both HAdVD-36 E4ORF1 and HAdVC-5 E4ORF1 transduced diet-induced obese mice [78]. In wild type mice transduced with a high dose of HAdVD-36 E4ORF1, *INSR* was downregulated, corresponding to an insulin-independent effect of E4ORF1 activity, and genes involved in glycolysis were upregulated, as was *PDK4*, an inhibitor of the TCA cycle [78]. Genes involved in gluconeogenesis were downregulated, as was *GYS2*, involved in the formation of glycogen [78]. *G6PD* was also downregulated [78]. Wild type mice transduced with a high dose of HAdVC-5 E4ORF1 did not have any differential expression of glycolytic genes, but some genes involved in lipid biosynthesis were upregulated [78]. In wild type mice transduced with low doses of HAdVD-36 E4ORF1, an increased prevalence of phospho-AKT and phospho-FoxO1 was observed [78]. Phospho-AKT induction was also observed in wild type mice transduced with a low dose of HAdVC-5 E4ORF1 [78]. Interestingly, and in contradiction with other literature [25,26], possibly due to tissue specific effects, MYC expression was not altered in wild type mice transduced with HAdVC-5 E4ORF1 [78]. To emphasize the importance of phospho-AKT for the anti-diabetic or anti-glycemic effects of HAdVD-36 E4ORF1, wild type mice treated with perifosine, an AKT inhibitor, did not show the decrease in blood glucose associated with HAdVD-36 E4ORF1 [78]. In summary, HAdVD-36 E4ORF1 was not only efficient at increasing the expression of metabolic genes in obese mice, but both HAdVD-36 E4ORF1 and HAdVC-5 E4ORF1 increased phospho-AKT (Table 3). This may represent a conserved mechanism by which E4ORF1 upregulates glycolysis across HAdV types.

## 7. E1A as a Regulator of Cellular Metabolism During Infection

Although E4ORF1 is the only HAdV protein with conclusive transcriptional effects on cellular metabolism, these studies suggest that at least one other HAdV encoded metabolic regulator exists [26]. The HAdV oncoprotein E1A has been shown to interact with a wide variety of host-cell proteins that are capable of influencing metabolism independently of an interaction with E1A [4,49,79,80,81]. In addition, because E1A is the first HAdV protein expressed during infection, it seems to be ideally positioned to establish early changes in cellular metabolism during HAdV infection.

Perhaps one of the first studies which suggested that E1A could influence cellular energy metabolism was performed in 1990 [82]. Expression of creatine kinase B, an enzyme responsible for maintaining cellular ATP levels [83], was shown to be induced by E1A [82]. This report represents the first suggestion that E1A may be responsible for inducing a cancer-like metabolic phenotype in human cells during infection. Another paper, published at roughly the same time, indicated that E1A was capable of inducing expression of thymidylate synthase, linking E1A to metabolic changes related to increased DNA synthesis [84].

A thorough metabolomic and transcriptomic study of IMR-90 cells transformed with E1A in conjunction with RAS revealed that glucose consumption and lactate secretion increased, as did glutamine consumption and glutamate secretion with transformation (Supplementary Table S1) [85]. The authors of this study elected to use E1A and RAS to study transformation as E1A alone only immortalizes, but does not transform, IMR-90 cells [85]. This is a caveat for the interpretation of this study towards the role of E1A in HAdV infection, as some of metabolic effects observed may mediated by RAS rather than E1A. Consumption and secretion of certain carboxylic acids and amino acids increased with transformation, as assayed from the extracellular media (Supplementary Table S1) [85]. A comparison of intracellular metabolites between E1A/RAS transformed IMR-90 cells versus wild type IMR-90 cells indicated that E1A/RAS transformed IMR-90 cells were much more metabolically active [85]. Intracellular glucose and pyruvate levels were lower in E1A/RAS transformed IMR-90 cells, as were concentrations of the amino acids (Supplementary Table S1) [85]. While lower intracellular concentrations of amino acids stand in contrast to what was observed in the context of wild type HAdVC-5 infection by Thai et al. [25], the increase in extracellular glutamine consumption is consistent with a number of papers examining metabolic changes due to HAdV infection [25,31]. Despite lower intracellular concentrations of amino acids, E1A/RAS transformed IMR-90 cells had increased amino acid consumption (Supplementary Table S1) [85]. Another indicator that E1A/RAS transformed IMR-90 cells were more metabolically active than wild type IMR-90 cells was the increase in the phosphocreatine to creatine ratio observed in transformed cells [85]. This ratio is a proxy for the cellular ATP/ADP ratio and energy state [85]. The higher metabolic activity of E1A/RAS transformed IMR-90 cells was further emphasized by the number of significant internal metabolite correlations within transformed cells [85]. There were 72 positive internal correlations and 92 negative internal correlations between the measured intracellular metabolites of E1A/RAS transformed IMR-90 cells, versus 23 positive internal correlations and 26 negative internal correlations in wild type IMR-90 cells [85]. The number of internal correlations is indicative of the number of perturbed metabolic pathways [86].

One of the unique correlations among metabolites upregulated in E1A/RAS transformed cells was a positive correlation between the levels of choline, involved in cell membrane structure [87], and the levels of the amino acids isoleucine, leucine, phenylalanine, tyrosine and lysine [85]. In addition, changes in phosphocholine, another component of cell membrane formation [87], was positively correlated with changes in isoleucine, leucine, phenylalanine, tyrosine and lysine [85]. It is unclear whether this increase in membrane metabolism components is specific to E1A/RAS transformed cells or is more widely applicable to HAdV infection, even though HAdV is a non-enveloped virus. However, choline consumption was increased in both HAdV infected HEK293 and 1G3 cells as discussed above (Supplementary Table S1) [31], which may point to an upregulation of cell membrane-component metabolism due to E1A in the context of HAdV infection.

Expression of genes involved in amino acid catabolism in the mitochondria, consistent with amino acid use as a significant energy source, were also upregulated in E1A/RAS transformed IMR-90 cells (Supplementary Table S2) [85]. Other genes encoding components of amino acid metabolism were similarly upregulated (Supplementary Table S2) [85]. In addition, genes involved in glucose metabolism were significantly increased in E1A/RAS transformed IMR-90 cells (Supplementary Table S2) [85]. Gene correlation analysis within E1A/RAS transformed IMR-90 cells indicated that expression of these amino acid catabolism genes, for example *BCKDHA*, were positively correlated with certain genes involved in the TCA cycle, such as *SUCLG1, IDH3B* and certain glycolytic genes, such as *ALDOC* [85]. In a number of ways, the phenotype observed with IMR-90 transformation by E1A/RAS follows the traditional definition of the Warburg effect, which is an upregulation of glycolysis and a downregulation of oxidative phosphorylation despite the presence of ample oxygen [13,14,15]. However, the increased consumption and potential utilization of amino acids as an energy source in E1A/RAS transformed IMR-90 cells indicate that oxidative phosphorylation through amino acid catabolism is another important metabolic pathway with nuanced regulation [85]. The increase in glycolysis and glutaminolysis occurring from E1A/RAS transformation is very similar to the increase in glycolysis and glutaminolysis attributed to the E4ORF1 protein of HAdV [25,26]. It is an interesting possibility that E1A contributes to cellular metabolic changes during HAdV infection in a manner similar to, but independent of, E4ORF1. If this question were to be examined, one confounding consideration would be that E1A is responsible for inducing transcription of E4ORF1, in addition to its role in modulating expression of many host-cell proteins during infection [79,88].

**Figure 6:**
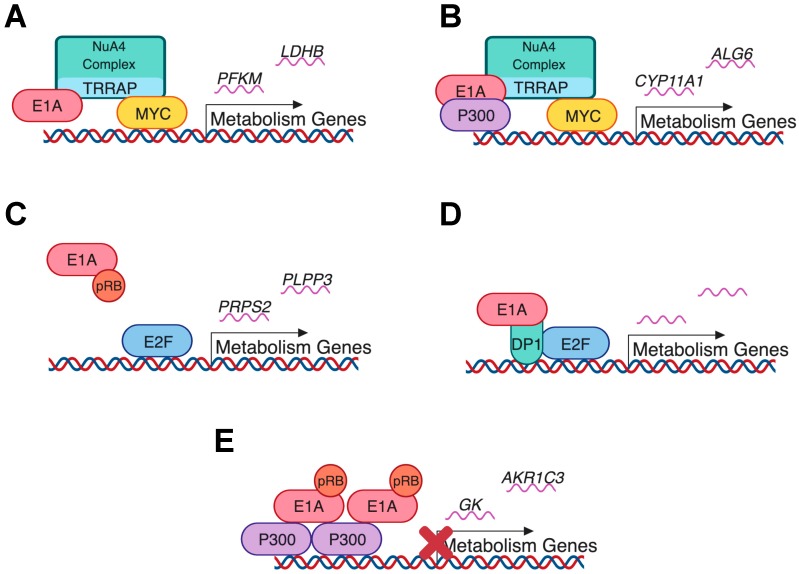
Putative mechanisms by which HAdV E1A regulates transcription of host-cell metabolic genes based on models derived from the literature [49,50,51]. (**A**) E1A can regulate metabolic gene expression through an interaction with the transcription factor MYC. E1A binds TRRAP, part of the NuA4 complex, which in turn is bound to MYC leading to increased transcription of metabolic genes [49]. *PFKM* and *LDHB* are two examples of transcripts that may be regulated due to this interaction based on supplementary data from Zhao et al. [49]. (**B**) The same paper indicated that E1A may be bound to p300 in addition to TRRAP and MYC leading to the expression of other E1A-regulated genes [49]. Again, *CYP11A1* and *ALG6* are two examples of metabolic genes potentially regulated by this interaction based on supplementary data from Zhao et al. [49]. (**C**) E1A can bind to pRB and release the inhibition of E2F-mediated gene transcription by pRB [51]. *PRPS2* and *PLPP3* are examples of two metabolic genes whose expression are decreased in a HAdVC-5 infection with a non-pRB binding E1A mutant compared to wild type infected cells and therefore could rely on the pRB-binding of E1A for expression during infection [51]. (**D**) E1A may also mediate the expression of E2F regulated genes through an interaction with DP1, which itself can bind to E2F and activate transcription [50]. No specific transcripts are shown, as this study by Pelka et al. did not include an RNA-seq component [50]. (**E**) Finally, an interaction between E1A, p300 and pRB may inhibit transcription of metabolism related genes through histone deacetylation [51]. *GK* and *AKR1C3* are two genes that may be regulated by E1A binding to p300 [51]. Image created with BioRender.

The interaction of E1A with host-cell proteins that can influence metabolism is also important when considering the role of E1A in host-cell metabolic reprogramming (Figure 6). Like E4ORF1, E1A is capable of influencing MYC activity [49]. However, this occurs indirectly via the interaction of E1A with the TRAAP protein of the NuA4 histone acetyltransferase complex, leading to increased transcription of MYC regulated genes (Figure 6A) [49]. An RNA-seq analysis of HS68 primary human foreskin fibroblast cells (Table 2) transduced with the TRRAP interacting region of E1A, indicated that 140 metabolic genes were upregulated, according to the supplementary data from that study [49]. An additional 92 metabolic genes were upregulated in conjunction with an interaction of E1A with p300 (Figure 6B), again extrapolated from the supplementary data of that paper [49].

In addition to the targets listed above, E1A can influence the E2F family of transcription factors via its interaction with their negative regulator Rb and Rb family members (Figure 6C) [89], or via a direct interaction with the DP-1 binding partner of the E2Fs (Figure 6D) [50]. It is well established that E1A sequesters Rb from E2F, leading to E2F activation. A study of transcriptional regulation by E1A indicated that the resulting E2F activation upregulates genes involved with RNA metabolism and biopolymer (macromolecule) metabolism in the host cell [90]. Additionally, E2F1 has been reported to influence oxidative phosphorylation and glycolysis [80]. Another comprehensive RNA-seq study of IMR-90 cells infected with a HAdVC-5 E1A mutant virus deficient for pRB binding, showed an upregulation of one metabolic gene, *TRIB1* (fold-change > 2) and a downregulation of approximately 89 metabolic genes (fold-change < 2) compared to wild type HAdVC-5 infected cells, as extrapolated from the supplementary RNA-seq gene list of that paper [51]. This suggests that pRB binding by E1A likely contributes to the regulation of these genes. This agrees with a high throughput study examining transcription factors potentially altered by HAdV infection, which found that E2F1 activity was increased [47].

Additionally, extrapolation of data from an RNA-seq analysis of IMR-90 cells infected with a HAdVC-5 E1A mutant virus deficient for p300 binding, indicated that 13 metabolic genes were upregulated (fold-change > 2) and 5 metabolic genes were downregulated (fold-change < 2) when compared to wild type HAdVC-5 infected cells [51]. Again, this suggests that the interaction of E1A with p300 (Figure 6E) modulates host-cell metabolism during infection. Due to the paucity of studies looking at metabolic effects of HAdV E1A, it seems that additional investigations of this area are clearly warranted. However, the interactions of E1A with MYC, pRB/E2F and p300 are an interesting starting point for understanding how E1A influences host-cell metabolism.

## 8. Conclusions

The changes in cellular metabolism enacted by HAdV infection very closely mimic the metabolic phenotype of cancer cells. HAdV infection induces an increase in glucose and glutamine consumption to fuel glycolysis and glutaminolysis, which ultimately lead to an increase in nucleotide production for DNA replication. However, advanced metabolomic techniques have added some nuances to this understanding, as the metabolic profile of infected cells is influenced by cell density and cell type. In addition, different HAdV types modulate metabolism by different mechanisms. Although this may occur primarily through the HAdV oncoprotein E4ORF1, it potentially also occurs through the HAdV oncoprotein E1A. Understanding how this altered metabolism contributes to HAdV replication may provide the insight needed to use small molecules that influence metabolism as anti-viral therapies. In light of a recent report that HAdV may be present in a number of diverse human tumours [12], understanding the metabolic modulation of HAdV may also have potential utility for cancer treatment.

## Figures and Tables

**Table 1:** Different HAdV species and associated types, tissue tropisms and clinically associated infections. The last column indicates whether the species contains the metabolism-associated *E4ORF1* viral gene.

Species	Types	Tissue Tropism (Types)	Associated Infections	Contains *E4ORF1* (Y/N)
A	12, 18, 31, 61	Gastrointestinal	Gastroenteritis	Yes
B	3, 7, 11, 14, 16, 21, 34, 35, 50, 55, 66, 68, 72, 79	Respiratory (3, 7, 16, 21, 50) Urinary/Renal (11, 14, 34, 35) Ocular (3, 7, 11, 14)	Acute respiratory disease, conjunctivitis, nephritis	Yes
C	1, 2, 5, 6, 57	Respiratory, Ocular (5)	Acute respiratory disease, conjunctivitis	Yes
D	8–10, 13, 15, 17, 19, 20, 22–30, 32, 33, 36–39, 42–49, 51, 53, 54, 56, 58–60, 62–65, 67, 69, 70, 71, 73–75, 81, 83–85, 90	Ocular, Gastrointestinal (36, 67)	Follicular conjunctivitis, pharyngeal conjunctival fever, epidemic keratoconjunctivitis, gastroenteritis	Yes
E	4	Respiratory, Ocular	Acute respiratory disease, conjunctivitis	Yes
F	40, 41	Gastrointestinal	Gastroenteritis	No
G	52	Gastrointestinal	Gastroenteritis	Yes
Unclassified/No record	76–78, 80, 82, 86–89	-	-	-

**Table 2:** Cell lines used by studies outlined in this review. Unless noted otherwise, all cell lines are human.

Cell Line	Donor Characteristics	Date Established	Cell Morphology	Tissue of Origin	Transformation Status
HeLa	Female—31 years old	1951 [32]	Epithelial	Cervical adenocarcinoma	HPV transformed
HEK ^1^	Fetus	1970 [33]	Epithelial	Embryonic kidney	Primary
HEK293	Female—Fetus	1977 [34]	Epithelial	Embryonic kidney	HAdV5 E1A transformed
1G3 ^2^	Fetus	2015 [35]	Amniocyte	Amniotic fluid	HAdV5 E1A transformed
IMR-90	Female—Fetus (16 weeks)	1977 [36]	Fibroblast	Lung	Primary
A549	Male—58 years old	1973 [37]	Epithelial	Lung adenocarcinoma	Transformed
SKOV3	Female—64 years old	1973 [38]	Epithelial	Ovarian adenocarcinoma ascites	Transformed
MCF10A	Female—36 years old	1990 [39]	Epithelial	Fibrocystic breast mammary gland	Spontaneously immortalized
NHBE	Human ^3^	N.A. ^3^	Epithelial	Bronchial	Primary
3T3-L1	Mouse—Fetus	1973 [40]	Fibroblast	Embryonic–pre-adipose	Spontaneously immortalized
BRK	Rat—Neonate	N.A. ^3^	Epithelial	Kidney	Primary
HS68	Newborn	1969 [41]	Fibroblast	Foreskin	Primary

^1^ Noted to be HeLa contaminated and is not the parent line of HEK293 cells; ^2^ Not to be confused with the mouse-derived hybridoma of the same name; ^3^ Batch-specific.

**Table 3:** Different metabolic effects in mice expressing E4ORF1 from HAdVD-36 or HAdVC-5 [78]. Diabetic mice (*db/db*) with transduced expression of HAdVD-36 E4ORF1 have decreased weight, increased glycemic control and glucose disposal. This is accompanied by decreased non-fasting blood glucose and adiponectin. In contrast, none of these effects, aside from decreased weight, occurred in diabetic mice with transduced expression of HAdVC-5 E4ORF1. Diet-induced obese mice with transduced expression of HAdVD-36 E4ORF1 display weight loss, increased glycolytic gene expression and lipid metabolism gene expression, while HADVC-5 E4ORF1 transduced mice only exhibit increased lipid metabolism. Wild type mice expressing HAdVD-36 E4ORF1 have decreased non-fasting blood glucose, increased glycolytic gene expression and increased phosphorylation of AKT and FoxO1. However, inflammation is also increased. Wild type mice expressing HAdVC-5 E4ORF1 do not show any changes in blood glucose levels, but do have increased metabolism gene expression and p-AKT. In contrast to other papers exploring the mechanism of HAdVC-5 E4ORF1 in culture [25,26], there were no changes in glycolytic gene expression or MYC activity in these animal studies (denoted by an asterisks *). Images created with BioRender.

	HAdVD-36 E4ORF1	HAdVC-5 E4ORF1
Diabetic mice 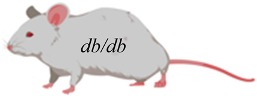	↑ glycemic control ↑ glucose disposal ↓ non-fasting blood glucose ↓ adiponectin ↓ weight ↓ lipid metabolism gene expression	∅ glycemic control ∅ blood glucose effects ↓ weight ↓ lipid metabolism gene expression
Diet-induced obese mice 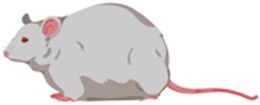	↑ glycemic control ↑ glycolytic gene expression ↑ lipid metabolism gene expression ↓ non-fasting blood glucose ↓ weight	↑ lipid metabolism gene expression ∅ glycemic control ∅ blood glucose effects ∅ weight change
Wild type mice 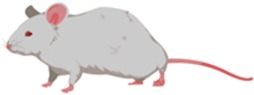	↓ non-fasting blood glucose ↑ glycolytic gene expression ↑ p-AKT and p-FoxO1 ↑ inflammation (high dose)	∅ blood glucose effects ∅ glycolytic gene expression * ↑ lipid metabolism gene expression ↑ p-AKT ∅ MYC activity changes *

## Supplementary Materials

The following are available online at http://www.mdpi.com/1999-4915/11/2/141/s1, Table S1: Metabolite Changes, Table S2: Metabolism Genes and Proteins.
